# Non-Targeted Metabolite Profiling Reveals Host Metabolomic Reprogramming during the Interaction of Black Pepper with *Phytophthora capsici*

**DOI:** 10.3390/ijms222111433

**Published:** 2021-10-22

**Authors:** Divya Kattupalli, Artur Pinski, Sweda Sreekumar, Aswathi Usha, Aiswarya Girija, Manfred Beckmann, Luis Alejandro Jose Mur, Soniya Eppurathu Vasudevan

**Affiliations:** 1 Transdisciplinary Biology, Rajiv Gandhi Centre for Biotechnology, Poojappura, Thiruvananthapuram 695014, India; divyak@rgcb.res.in (D.K.); swedasreekumar@rgcb.res.in (S.S.); aswathiu@rgcb.res.in (A.U.); 2 Plant Cytogenetics and Molecular Biology Group, Institute of Biology, Biotechnology and Environmental Protection, Faculty of Natural Sciences, University of Silesia in Katowice, 40-032 Katowice, Poland; artur.pinski@us.edu.pl; 3 Institute of Biological, Environmental and Rural Studies, Aberystwyth University, Ceredigion SY23 3EE, UK; aig15@aber.ac.uk (A.G.); meb@aber.ac.uk (M.B.)

**Keywords:** *Piper nigrum*, quick wilt, metabolomics, abscisic acid, salicylic acid, jasmonic acid

## Abstract

*Phytophthora capsici* is one of the most destructive pathogens causing quick wilt (foot rot) disease in black pepper (*Piper nigrum* L.) to which no effective resistance has been defined. To better understand the *P. nigrum*–*P. capsici* pathosystem, we employed metabolomic approaches based on flow-infusion electrospray–high-resolution mass spectrometry. Changes in the leaf metabolome were assessed in infected and systemic tissues at 24 and 48 hpi. Principal Component Analysis of the derived data indicated that the infected leaves showed a rapid metabolic response by 24 hpi whereas the systemic leaves took 48 hpi to respond to the infection. The major sources of variations between infected leaf and systemic leaf were identified, and enrichment pathway analysis indicated, major shifts in amino acid, tricarboxylic acid cycle, nucleotide and vitamin B6 metabolism upon infection. Moreover, the individual metabolites involved in defensive phytohormone signalling were identified. RT-qPCR analysis of key salicylate and jasmonate biosynthetic genes indicated a transient reduction of expression at 24 hpi but this increased subsequently. Exogenous application of jasmonate and salicylate reduced *P. capsici* disease symptoms, but this effect was suppressed with the co-application of abscisic acid. The results are consistent with abscisic acid reprogramming, salicylate and jasmonate defences in infected leaves to facilitate the formation of disease. The augmentation of salicylate and jasmonate defences could represent an approach through which quick wilt disease could be controlled in black pepper.

## 1. Introduction

Black pepper (*Piper nigrum* L.) is an important spice crop of southern India and in many tropical regions where it is of major economic relevance [1,2]. Significant reduction in the production of black pepper is mostly due to a wide range of destructive diseases. The most devastative and widespread disease that affects peppercorn production is Phytophthora foot rot (‘quick wilt’) caused by the filamentous oomycete, *Phytophthora capsici.* It is an endogenous oomycete that initially affects the collar region and gradually destroys the entire plant [3,4]. Most biocides are poorly effective towards *P. capsici* infection moreover, this type of control may lead to the evolution of resistant strains. To date, no resistance mechanisms have been characterised in black pepper against quick wilt disease [5].

The formation of disease in plants depends, to a large extent on the pathogen either avoiding interaction with a series of host-encoded receptors or neutralizing the host immune responses [6,7]. Pattern recognition receptors will interact with various pathogen-associated molecular patterns (PAMP) to elicit PAMPs-triggered immunity (PTI) [7]. During infection, the PTI is suppressed by pathogen-encoded cytoplasmic or apoplastic effectors [8]. These effectors are recognized by plant-encoded nucleotide-binding site leucine-rich repeat (NBS-LRR) resistance (*R*) genes, leading to effector-triggered immunity (ETI) often leading to the formation of a programmed cell death known as the hypersensitive response (HR) [9]. Such mechanisms were demonstrated in other oomycetes like *Phytophthora infestans*, where the well-characterised RXLR (Arg-x-Leu-Arg) effectors suppress host immunity [10] by targeting the susceptibility (S) factors [11]. Furthermore, *P. capsici* effectors can also activate reactive oxygen species (ROS)-mediated cell death, most likely by degrading proteins binding to Accelerated cell death 11 (ACD11) [12]. ACD11 cell death depends on the defensive phytohormone, salicylic acid (SA) [13]. Similar studies highlight the influence of phytohormones in disease resistance as well as the successful establishment of the pathogen in plants.

SA was first characterised as a contributor to systemic acquired resistance (SAR) but is also involved in the regulation of PTI and ETI. Jasmonates (JA) function along with ethylene (ET) in induced systemic resistance, but also have roles in defence against pathogens where host cell death is initiated as part of the pathogenic process, i.e., necrotrophs [14,15,16,17]. Abscisic acid (ABA) has equivocal roles in plant defence and can act either by increasing susceptibility or conferring resistance to the host, depending on circumstances. For example, ABA-treated plants showed improved resistance against *Alternaria solani* infection in tomato seedlings and *Linum usitatissimum* plants towards *Fusarium oxysporum* [18,19]. In contrast, ABA increases the susceptibility of Arabidopsis to *Pseudomonas syringae* pv. *tomato* [20] and of rice to the rice blast pathogen, *Magnaporthe grisea* [21]. ABA appears to suppress SA-mediated SAR [22] and other SA-events via modulation of the SA biosynthetic gene *Isochorismate synthase1* (*ICS1*) [23] or SA signalling via (e.g.,) NPR1 [21]. Within an oomycete pathogen context, suppression of ABA synthesis in potato enhanced defence against *P. infestans* [24]. ABA can contribute to defence by initiating ROS or nitric oxide production [25,26] and also prime herbivore-initiated induced systemic resistance [27]. Thus, the role of phytohormones is likely to be context-specific, varying with the type of plant-pathogen interaction.

Our understanding of plant-pathogen interactions can be enhanced by metabolomic approaches which allow the nonbiased, high-throughput examinations of biochemically complex plant extracts [28]. Metabolomic approaches have been used to track metabolic responses during pathogen attack in model systems such as Arabidopsis to describe *Pseudomonas syringae* infection [29], and in *Brachypodium distachyon* to *Magnaporthe grisea* infection [30]. Global metabolite profiling of wild type *Fusarium graminearum* and Δ*tri5* mycotoxin mutant infected wheat described differential metabolic reprogramming in the rachis nodes of wheat heads [31]. Metabolomics has also highlighted the roles of amino acids and phenolics in defence against *Rhizoctonia solani* in rice [32] and maize [33]. Additionally, metabolomics can also provide biomarkers for infections. For example, tomatidine, saponins and isocoumarins were identified as biomarkers for *P. infestans* infected tomato [34]. Moreover, the cross-species ubiquity of many metabolites allows metabolomics to be applied to any plant species, not just model plants. Metabolomics, therefore, represents a powerful approach to assess defence metabolism in plants.

Our work has previously focused on examining the potential roles of small non-coding RNAs (sncRNA) in quick wilt disease development. We have examined miRNA-mediated gene regulation in *P. nigrum* [35], tRNA-derived sRNAs effects on the transcriptome of pathogen-infected plants [36] and the role of microRNA mediated post-transcriptional regulation during *P. capsici* infections. Moreover, our studies in black pepper during *P. capsici* infection demonstrated the differential regulation and the presence of phytohormone-responsive transcription activators in the promoter regions of *PR-1* genes, which are crucial for SAR induction during pathogen attack [37]. In our current study, we employ flow-infusion electrospray-high-resolution mass spectrometry (FIE-HRMS) to provide a comprehensive description of the metabolome during quick wilt disease development, both at the site of infection and systemically. Disease development was associated with changes in amino acid metabolism, the TCA cycle and vitamin B6 pathways. Crucially, impacts on SA and JA biosynthesis and ABA responses were also seen. The results obtained from this study reveal the dynamic role of host reprogramming during *P. capsici* infections of black pepper.

## 2. Results

### 2.1. Metabolic Changes in P. nigrum Leaves during P. capsici Infection

**Figure 1:**
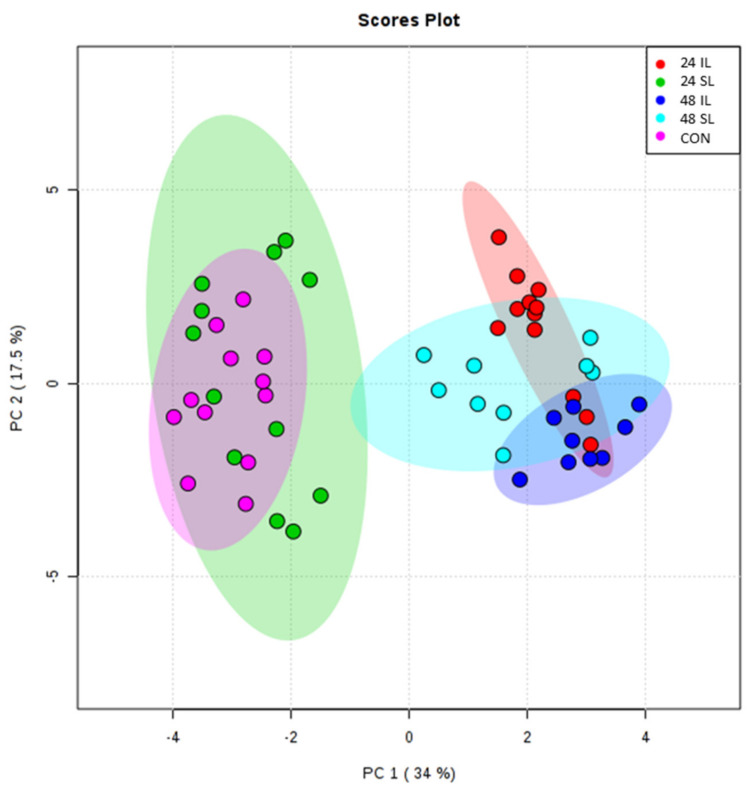
Principal Component Analysis of *Phytophthora capsici* challenged Black Pepper (*Piper nigrum* L.) leaves. Leaves were sampled at the site of infection (IL) and in the leaf immediately above the infection site (systemic, SL) at 24 and 48 h after the challenge. Changes were compared to control; mock-inoculated leaves (CON). Larger ovals represent 95% confidence intervals for each group.

Infected leaves (‘IL’) and systemic leaves (‘SL’) were sampled at 24 and 48 hpi (hours post-inoculation) following challenge with *P. capsici* and control leaves (CON) from mock-inoculated plants, were used for non-targeted metabolomic profiling using FIE-HRMS. Overall metabolic variation was visualized using principal component analysis (PCA) (Figure 1). This indicated that there were significant differences in the metabolites of IL compared to CON samples but with SL samples, differences were only seen at 48 hpi. The sources of variation (*m*/*z*) across principal component 1 (PC1), which related to responses linked to infection, were targeted using ANOVA (*p* ≤ 0.05). Individual *m*/*z* were identified based on accurate mass values and ionization patterns.

**Figure 2:**
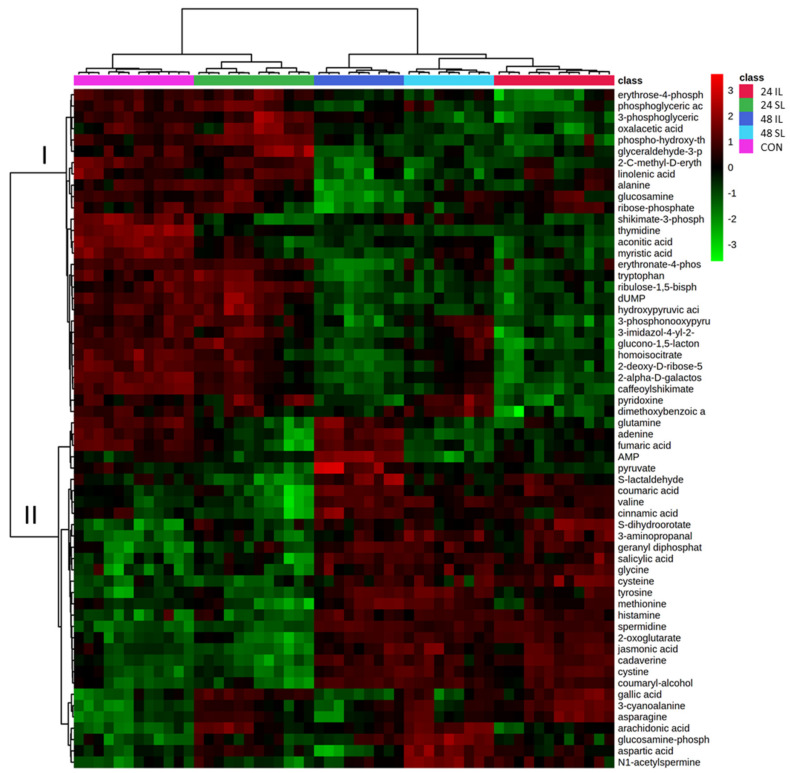
Heat map showing the major sources of variation in the metabolome Black Pepper (*Piper nigrum* L.) leaves challenged with *Phytophthora capsici*. Metabolites exhibiting significant variation in leaves were samples at the site of infection (IL) and in the leaf immediately above the infection site (SL, systemic leaf) at 24 and 48 h after infection and controls (CON) were targeted by ANOVA. The relative accumulation of each is displayed using a heat map. The heat map was divided into two clades: In clade I, metabolites in this category included TCA metabolites such as oxaloacetate, aconitic acid, ribulose-bisphosphate etc. Clade II contains metabolites including defence phytohormones (SA and JA), amino acids (valine, glycine, cysteine, tyrosine, methionine, histamine, glutamate) and amides (cadaverine, spermidine) metabolites.

The metabolites and their accumulation patterns are shown in Figure 2. The heatmap indicated that levels of statistically significant metabolites in 24 IL, 48 IL and 48 SL samples were distinct to CON, but not the 24 SL samples. Two broad metabolite accumulation patterns could be distinguished as indicated by two clades (I and II) in Figure 2. In clade I, metabolites are relatively high in CON (and 24 SL) and were lower as the infection progressed. Metabolites in this category included TCA metabolites such as oxaloacetate, aconitic acid, ribulose-bisphosphate etc. In clade II, metabolites were low in CON (and 24 SL) but increased with disease development. Clade II metabolites included defence phytohormones (SA and JA), amino acids (valine, glycine, cysteine, tyrosine, methionine, histamine, glutamate) and amides (cadaverine, spermidine). Interestingly, the TCA metabolite, fumarate, was placed in clade II but showed increased accumulation only at 48 IL.

**Figure 3:**
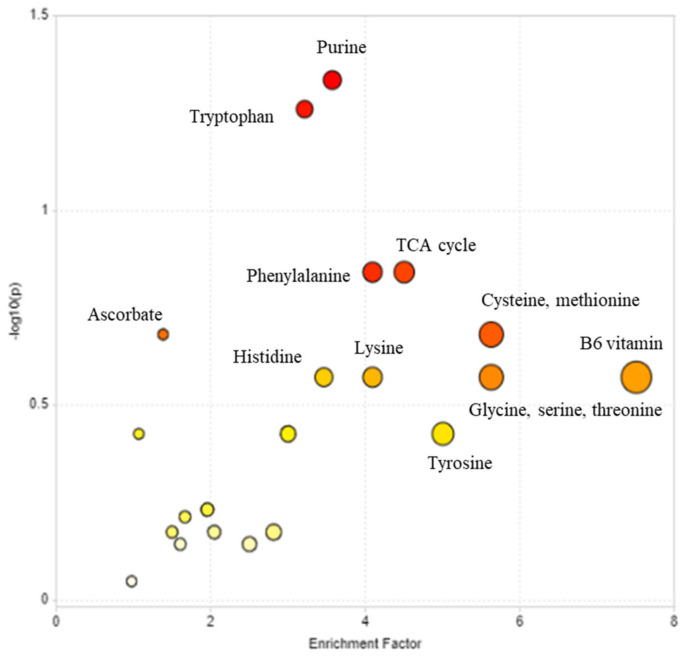
Pathway enrichment analysis of metabolites varying in the metabolome of Black Pepper (*Piper nigrum* L.) leaves challenged with *Phytophthora capsici*. Metabolites exhibiting significant variation in leaves (Figure 2) were assessed for pathway enrichment. Pathways showing the highest enrichment and metabolomic impact are plotted.

To ease comprehension of the metabolomic changes, the targeted metabolites were assessed for pathway enrichment analyses (Figure 3). The analysis indicated a wide-ranging change in amino acid metabolism, with pathways involved including cysteine, methionine, lysine, phenylalanine, tryptophan, histidine, glycine, serine, and threonine metabolism. In addition to those, significant changes were noted in the TCA cycle and purine metabolism. The variables were also mapped onto KEGG global metabolite pathways (Figure S1). This illustrated the changes in nucleotide, amino acid and bioenergy pathways. Figure S1 also highlighted changes in the B6 vitamin pathway and linoleate metabolism; the latter possibly linked to JA biosynthesis. Amino acids and TCA/bioenergy metabolite changes were examined in separate heat maps (Figure S2). Shifts in amino acids in IL were observed from 24 hpi. Serine, tyrosine, tryptophan, proline, valine, leucine, phenylalanine, glutamate, glutamine, methionine, asparagine and aspartic acid increased with disease development (Figure S2A). SL showed similar changes at 48 hpi. The exception was lysine which showed a significant reduction in the infected leaves compared to control. One possible explanation in the low levels of lysine could be related to piperine biosynthesis (https://www.genome.jp/kegg-bin/show_pathway?ko00960 accessed on 19 March 2021), but other metabolites in this pathway did not significantly differ on infection. When all TCA metabolites were displayed on a heatmap, no patterns were observed that could be linked to infection (24 IL, 48 IL) (Figure S2B).

**Figure 4:**
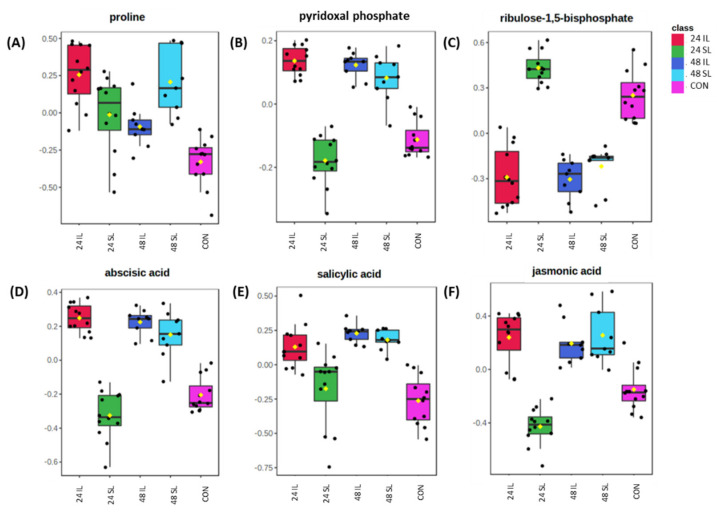
Metabolites varying in Black Pepper (*Piper nigrum* L.) leaves challenged with *Phytophthora capsici.* Key exemplar metabolites exhibiting significant variations (**A**) Proline (**B**) pyridoxal phosphate (**C**) ribulose-1,5-bisphosphate (**D**) abscisic acid (**E**) salicylic acid and (**F**) jasmonic acid in *Piper nigrum* control leaf (CON), *Phytophthora capsici* infected *Piper nigrum* infected leaves (IL) and leaf immediately above the infection site-systemic leaves (SL) at 24 h and 48 h.

Individual metabolites of particular biological relevance were displayed in box and whisker plots (Figure 4). Proline is often associated with stress responses [38] and this was significantly elevated in all infected tissues compared to CON (Figure 4A). Vitamin B6 metabolism was prominent in the enrichment analysis and the active vitamin B6 form, pyridoxal phosphate, showed significant changes, with a rapid increase in IL tissues and increased in 48 SL (Figure 4B). Infections with *P. capsici* led to the formation of a blackened lesion which, presumably, perturbs photosynthesis which could be linked with decreases in the carbon assimilate ribulose-1, 5-bisphosphate in 24 IL and 48 IL samples compared to CON and 24 SL (Figure 4C). However, a similar decrease was seen for ribulose-1, 5-biphosphate in 48 SL when leaves appeared symptomless, suggesting that disease symptoms were rapidly being initiated systemically. The level of ABA increased in 24 IL and this increase in ABA levels was also seen in 48 IL and 48 SL (Figure 4D). This pattern of increase was also seen with other defence-associated phytohormones SA and JA (Figure 4E,F).

### 2.2. Assessing Defence Hormone Interactions during P. capsici Infections

**Figure 5:**
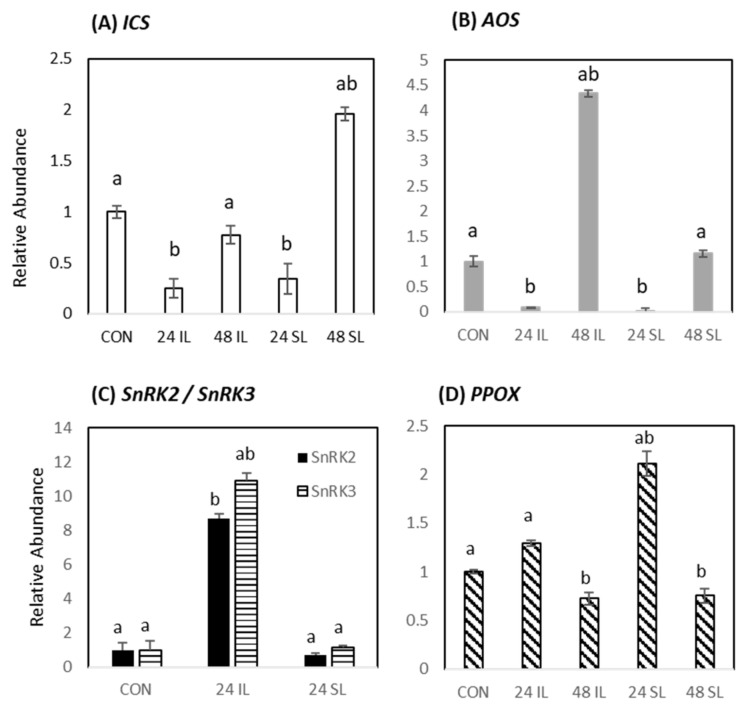
RT-qPCR assessment of key pathways suggested by metabolomic assessments of Black Pepper (*Piper nigrum* L.) leaves challenged with *Phytophthora capsici*. Expression of (**A**) the salicylic acid biosynthetic gene—*isochorismate synthase1* (*ICS1*) (**B**) the jasmonate biosynthetic gene—*allene oxide synthase* (*AOS*), (**C**) the ABA-responsive *snf1-related protein kinase 2* and *3* (*SnRK2*/*SnRK3*), (**D**) Vitamin B6 biosynthetic gene—*pyridoxamine 5′-phosphate oxidase* (*PPOX*) and in the leaf immediately above the infection site (SL, systemic leaf) at 24 and 48 h after challenge and controls (CON). Statistical significance was determined by performing an ANOVA, statistically significant values (*p*-value ≤ 0.05) were shown with different letters, whereas insignificance was labelled with the same letters.

To independently verify some of the findings from the metabolomic studies, RT-qPCR of some candidate key genes in targeted biosynthetic pathways was undertaken. From the metabolic data, we found that the levels of phytohormones increased significantly in infected leaves compared to control. To confirm the SA and JA changes, the expression of key genes involved in the biosynthesis of corresponding phytohormones were assessed, namely *ICS1* for SA [39] and allene oxide synthase (*AOS*) for JA [40]. ABA associated changes were indicated by expression patterns of the ABA-responsive *Snf1-related protein kinase2* and *3* (*SnRK2, SnRK3*). The relative expression of these genes in the infected and control leaf samples was calculated in comparison to the endogenous gene, actin (Figure 5). The expression of *ICS1* was significantly (*p* < 0.05) reduced in both IL and SL tissue at 24 hpi. By 48 hpi, *ICS1* expression of IL was similar to those of CON tissue but in SL tissue *ICS1* levels were increased at 48 hpi (Figure 5A). *AOS* showed a similar expression pattern and showed a transient suppression in both IL and SL at 24 hpi but this was followed by a significant increase in expression over CON in IL at 48 hpi (Figure 5B). These patterns were consistent with a systemic reduction in SA and JA biosynthetic gene expression during the early stages of infection (24 hpi) but recovered by 48 hpi. When compared to SA and JA metabolite levels, this did not suggest a close correlation with biosynthetic gene expression (Figure 4) where a reduction in metabolite levels was not seen in IL and SL at 24 hpi. Very significant increase in *SnRK2* and *SnRK3* expression in 24 IL samples suggested, ABA responses were prominent at the site of infection but no changes were observed systemically (Figure 5C). This implied that any ABA suppressive effect on SA and JA would only be relevant locally at 24 hpi. Transcriptional changes in the vitamin B6 biosynthetic pathway were assessed by measuring the expression of pyridoxamine 5′-phosphate oxidase (*PPOX*) (Figure 5D). This showed a significant increase only in SL tissue at 24 hpi.

**Figure 6:**
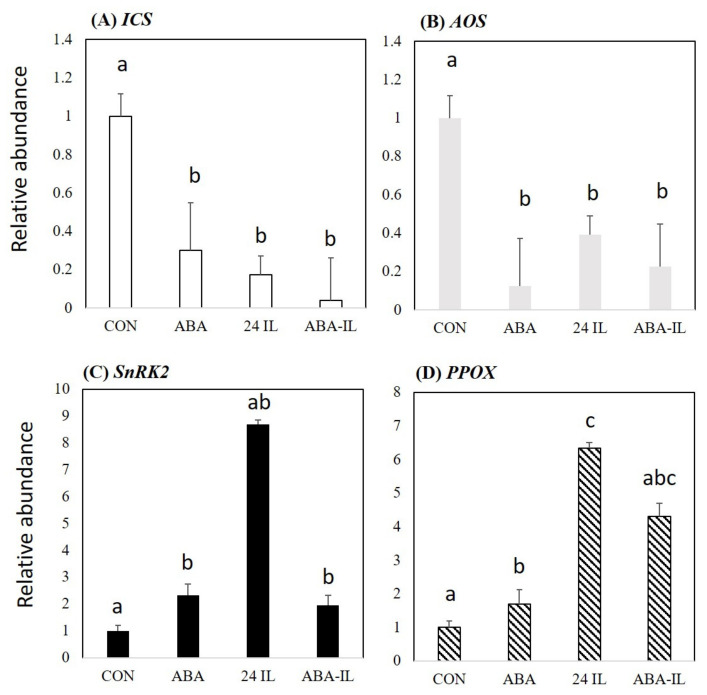
RT-qPCR assessment of ABA effects on key pathways suggested by metabolomic assessments of Black Pepper (*Piper nigrum* L.) leaves challenged with *Phytophthora capsici*. Expression of (**A**) the salicylic acid biosynthetic gene—*isochorismate synthase1* (*ICS1*) (**B**) the jasmonate biosynthetic gene—*allene oxide synthase* (*AOS*), (**C**) the ABA-responsive *snf1-related protein kinase 2* (*SnRK2*), (**D**) the vitamin B6 biosynthetic gene—*pyridoxamine 5′-phosphate oxidase* (*PPOX*) in controls (CON), at 24 h following treatment with 100 µM ABA (ABA), 24 h post inoculation (24 IL) and 100 µM ABA treated leaves that had been infected with *P. capsici* at 24 h (ABA-IL). Statistical significance was determined by performing an ANOVA, statistically significant values (*p*-value ≤ 0.05) were shown with different letters, whereas insignificant were labelled with the same letters.

To test whether ABA has a suppressive effect on the defence response, we examined the effect of exogenous application of ABA on the *P. nigrum*–*P. capsici* interaction. Application of 100 µM ABA to *P. nigrum* plants, reduced expression of *ICS1* and *AOS* at 24 hpi following treatment compared to CON (Figure 6). Examining the expression of the ABA-responsive gene *SnRK2* confirmed that exogenous application was eliciting a response from the plant, although this was not as prominent as in 24 IL samples. Interestingly, in samples that were both infected and ABA treated (ABA-IL), the levels of *SnRK2* expression were similar to those of ABA application alone. This could imply a dose-dependent effect of ABA on *SnRK2* expression. Our assessment of *PPOX* expression indicated that this was increased by 100 µM ABA treatment compared to CON. Infection with *P. capsici* also elevated *PPOX* (24 IL) expression, which could reflect an endogenous increase in ABA but, *SnRK2* expression in ABA-IL samples was reduced compared to 24 IL. This effect of concatenated endogenous and exogenous ABA could also suggest a dose-dependent effect on *PPOX* expression.

**Figure 7:**
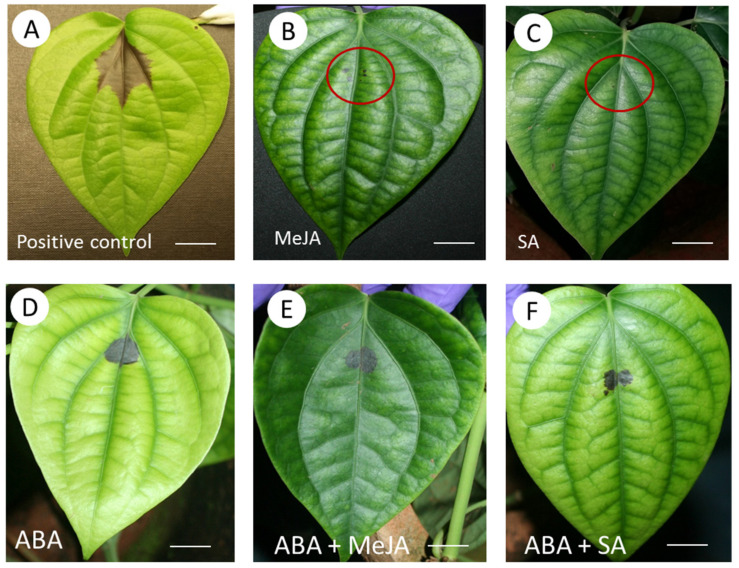
Phenotypes in Black Pepper (*Piper nigrum* L.) leaves at 24 h challenged with *Phytophthora capsici* and treated with (**A**) water, (**B**) 0.5 mM methyl jasmonate (MeJA), (**C**) 1 mM salicylic acid (SA), (**D**) 100 μM abscisic acid (ABA), (**E**) ABA + MeJA and (**F**) ABA + SA. The infection site is circled in red in some cases. Bar = 1 cm.

To further determine the impact of SA and JA during quick-wilt disease development, we exogenously applied SA and JA. *P. nigrum* leaves were pre-treated with 1 mM SA or 500 µM MeJA and then challenged with *P. capsici*. The concentrations used reflected the ranges commonly used for these phytohormones when exogenously applied. Compared to CON, untreated, *P. capsici* infected plants, SA and JA pre-treated leaves showed symptoms that were restricted to the wound site added experimentally to aid the infection process (Figure 7). However, when 100 µM ABA was co-applied with either SA or JA, the leaves exhibited black lesions at the infection site. This suggested that SA and JA mediated resistance was at least partially reversed by ABA treatment.

## 3. Discussion

Black pepper (*P. nigrum*) is an important spice crop and benefits the Indian economy. One of the major concerns in black pepper production is the yield losses due to quick wilt disease. A common strategy used to combat disease is the development of resistant germplasm. However, to date, no suitable tolerant *P. nigrum* germplasm has been defined and most biocides are poorly effective [5]. This reflects a lack of information on the progression of quick wilt disease and the defence mechanism in black pepper. Here, we have used untargeted metabolomic approaches to assess the metabolite changes during quick wilt disease development.

### 3.1. The Early Stages of Quick Wilt Disease Involve Changes in Primary Metabolism

During adverse conditions, plants shift metabolism to influence growth, development, and particularly immunity [41,42,43]. Equally, these changes can provide nutrients to the pathogen and aid in disease progression [44]. Thus, the outcomes of plant-pathogen interactions were modulated by either synthesizing [42] or degrading [45] carbohydrates, amino acids and lipids associated with primary metabolism. Such changes in primary metabolism were also observed in the interaction of *P. nigrum* and *P. capsici*, especially amino acid, TCA cycle, nucleotide and vitamin B6 metabolism.

Amino acids play a key role in plant-pathogen interactions and act as precursors for the biosynthesis of defence compounds such as phytoalexins [46,47]. This implies that the changes in primary metabolites that we observed were largely occurring in the plant and not in the pathogen [48]. The acidic amino acid glutamate is a major N-assimilatory metabolite that forms the basis for synthesising key defence-related metabolites such as proline, arginine and γ-aminobutyric acid (GABA) [49,50,51]. In the interaction of *P. capsici* with black pepper, glutamate accumulation was observed, especially at later infection time points. Such changes would feed into the amino acid biosynthetic pathways which were enhanced in the metabolomes. However, the accumulation patterns of different amino acids were very variable, occurring in IL and SL at different time points. It may be that these changes reflect altered patterns of protein synthesis as the disease develops.

Plant responses to pathogens are energy demanding processes that involve reprogramming towards defence or providing nutrients for the invader [52,53]. Much of this energy demand is met by the TCA cycle [41,54]. Therefore it was relevant that the TCA cycle was one of the enriched pathways following the challenge with *P. capsici* and was prominent in the KEGG map of major sources of variation. Bioenergetic metabolism can also provide the carbon skeletons to both amino acid and nucleotide biosynthesis and both were seen in responses to *P. nigrum* and *P. capsici*. However, our comparison of individual TCA metabolites suggested variable accumulation patterns so that no clear trend linked to infection could be seen. Interestingly, we did not observe changes in sugar metabolism in our experiment, although we observed reductions in ribulose-1,5-bisphosphate which is a likely indicator of lower photosynthetic activity. This also did not align with the changes seen in Arabidopsis following the challenge with *Pseudomonas syringae* pv. *tomato* (*Pst*) where differences in sugars were observed [29]. This could reflect different effects following infection with very different plant pathogens and/or the influence of the host.

Altered primary metabolism in the interaction of *P. nigrum* and *P. capsici* is further implied from another enhanced pathway, B6 (pyridoxine) metabolism. Vitamin B6, as pyridoxal 5′-phosphate, is an important coenzyme in amino acid, glucose, and lipid metabolism [55]. In vitamin B6 metabolism, pyridoxine 5′-phosphate (PNP) and pyridoxamine 5′-phosphate (PMP), can be converted into pyridoxal 5′-phosphate by the oxidase PDX3 [56]. PMP/PNP oxidase PDX3 is a salvage pathway enzyme essential for balancing B6 vitamer levels in Arabidopsis. *Pdx3* mutants exhibit PMP accumulation and compromised nitrogen metabolism [57] but also up-regulated defence-related genes [58]. Within the context of plant stress, vitamin B6 is a ROS scavenger that increases resistance to biotic and abiotic stresses and can be part of a coordinated antioxidant response to infection, for example, to *Erwinia carotovora* subsp. *carotovora* [59,60]. However, in *N. tabacum* increasing the levels of pyridoxine delays and reduces plant defences linked to the HR and increases the severity of disease symptoms [61]. In our *P. nigrum*–*P. capsici* interaction the increase in pyridoxal 5′-phosphate could not be linked to changes in antioxidant metabolism as, for example, ascorbic acid was not a major source of variation in our datasets. As the Colinas and Fitzpatrick study [58] implicated vitamin B6 metabolism as a suppressor of SA production, the up-regulation of the vitamin B6 pathway could be a feature of how *P. capsici* infection reprograms the metabolism to neutralize host defence.

### 3.2. SA, JA and ABA as the Major Factors in Defence Regulation in P. nigrum

The evolution of pathogens is highly sophisticated and includes complex molecular mechanisms to either modulate the biosynthesis of phytohormones and/or perturb hormonal signalling pathways [62,63]. Secondary metabolites such as SA, JA and ET serve as key regulators of plant resistance responses [64]. Thus, it could be expected that SA and JA levels either do not change or are suppressed following the challenge with *P. capsici*. Indeed, the transcription of the *ICS1* and *AOS*, which are important regulators of SA and JA biosynthetic pathways, respectively, were reduced in *P. capsici* challenged *P. nigrum* compared to the controls. However, this was not the result seen with the metabolomic assessment of *P. nigrum* challenged with *P. capsici* which suggested that both SA and JA levels were increasing on the infection. These contradictory results could indicate that these increases in SA and JA were not arising from de novo synthesis but through the other pre-existing stores. For SA, these could arise from glucosylated [65] or hydroxylated [66] forms or, with JA, from glycosyl esters and amino-linked conjugates [67]. These changes were not seen in the metabolome profiling as such processing could involve the production of many conjugates, which could be below the detection limit of FIE-HRMS. Whatever the sources of SA and JA, their role in the developing quick wilt disease required consideration. We demonstrated that SA and JA could be effective in *P. nigrum* as the exogenous application of either hormone could suppress the development of quick wilt symptoms. Therefore, the observed increase in endogenous SA and JA with *P. capsici* infection could present a ‘failed’ defence response which may be part of a reprogramming towards reduced defences. There are precedents for this in the literature with key components of SA-mediated defences such as the signalling molecule NPR1, pathogenesis-related proteins and SA binding proteins being suppressed by pathogens [68]. Mechanistically, this could involve the well-described SA–JA signalling antagonism [64]. For example, *Pseudomonas syringae* produces the JA-mimicking phytotoxin coronatine, which enhances virulence by reducing effective SA-dependent responses in Arabidopsis [69]. Conversely, SA suppresses JA signalling downstream of the SCF(COI1)-JAZ receptor complex by reducing the accumulation of the GCC-box binding transcription factor ORA59 [70]. Away from model systems, *Marchantia polymorpha*, a thalloid liverwort, develops infection of *Irpex lacteus* on treatment with SA, while the effect was suppressed when co-treated with the bioactive jasmonate, dn-cis-OPDA, suggesting antagonistic interactions between SA and oxylipin pathways [71]. It should be noted that dependent on relative concentrations, SA and JA can also act synergistically to boost defences [72].

Beyond SA–JA antagonism, ABA can play a key modulatory role in defence. For example, *P. syringae* pv. *tomato* was suggested to inhibit resistance mediated by the resistance gene, *RPS2*, through ABA-mediated repression of the NPR1-dependent defence pathway [73]. Further, SAR, mediated by SA, can be suppressed by ABA in Arabidopsis [23]. This can be achieved by suppressing the accumulation of defence associated lignin [74], callose [75], nitric oxide [76] and other defence gene expression [74]. In contrast, ABA biosynthetic mutants exhibit enhanced resistance to *P. syringae* [77]. ABA acts antagonistically to SA signalling by compromising the rice resistance to *Xanthomonas oryzae* pv *oryzae* [78] and *Magnaporthe grisea* [21]. ABA–PYR1 receptor activity represses the SA-mediated signalling which is required for resistance to biotrophic pathogens [79]. ABA signalling also inhibits necrotrophic interactions by interfering with SA/JA/ET-mediated signalling in Arabidopsis infected with *Plectosphaerella cucumerina* [80]. This suppressive ABA activity may be mediated through the PYR1 component of PYR/PYL/RCAR ABA receptors to suppress *SnRK2* protein kinases [79]. ABA seems likely to be playing a similar role in the black pepper—*P. capsici* system. There was clear evidence of ABA accumulation occurring rapidly following *P. capsici* infection*,* with resulting changes in *SnRK2* and *SnRK3* gene expression and exogenous application that could reverse SA–JA boosted defence. Crucially, when we co-applied ABA with JA or SA, disease symptoms were again observed. These results implicate ABA as functioning to counter the effects of SA and JA, at least in the first 24 hpi to establish infection in *P. nigrum*.

## 4. Materials and Methods

### 4.1. Plant Material, Culture and Infection

*P. nigrum* (ecotype Panniyur I) plants and the virulent *P. capsici* strain was provided by the Department of Plant Pathology, College of Agriculture, Thiruvananthapuram, India. The plants were maintained as described in Asha et al. [35]. *P. capsici* inoculation protocols were done as described by Anith et al. [81]. *P. capsici* was sub-cultured on Potato Dextrose Agar (PDA) every two weeks. Healthy four tofive-week-old plantlets were selected, and the second youngest leaf was infected with *P. capsici* mycelial discs on the abaxial side. Control samples consisted of mock-inoculations with PDA alone. Infected leaves (IL) and the leaves immediately above the infected leaf which is denoted as the systemic leaf (SL) were sampled from the infected plantlets post 24 hpi and 48 hpi. Control (CON) leaves were collected from an equivalent site on the uninfected plantlets. All the experiments were carried out in triplicates.

### 4.2. Metabolite Extraction for FIE-HRMS

About 1 g of leaves from control and infected plantlets were flash-frozen in liquid nitrogen and were either immediately extracted or stored at −80 °C until processing. The samples were ground with liquid N2 using mortar and pestle. Four biological replicates were taken for every experimental class. From that three sub-samples per leaf of about 100 mg were taken and considered as technical replicates (4 × 3 = 12). Metabolites were extracted in 1 mL chloroform/methanol/water (1:3:1 *v*/*v*/*v*) solution and shaken for 30 min at 100 rpm in a 4 °C shaker. After that, the samples were centrifuged at 6000× *g* for 1 min at 4 °C. The supernatant was collected and dried in a vacuum concentrator without heating. After drying, 200 μL of 50% methanol was added and 70 μL was transferred into an HPLC glass vial with a 0.2 mL flat-bottom micro insert. Samples were run randomly using an autosampler with a tray temperature of 15 °C. The sample injected was set at a volume of 20 μL into a flow volume of 60 μL/min water in 70% water: 30% methanol, using a surveyor liquid chromatography system (Thermo Scientific, Waltham, MA, USA). FIE-HRMS was performed using an Exactive Plus Orbitrap MS (Thermo Scientific, Waltham, MA, USA). Mass-ions (*m*/*z*), were generated in both positive and negative ionization modes over four scan ranges (15–110, 100–220, 210–510, 500–1200 *m*/*z*) with an acquisition time of 5 min (Supplementary Table S1). Individual ionization peaks values were normalized as a percentage of the total ion count for each sample. The resulting high-resolution accurate mass values were used to interrogate the KEGG (Kyoto Encyclopedia of Genes and Genomes) database (http://www.genome.jp/kegg/ accessed on 15 January 2021) and Direct Infusion Metabolite database (https://dimedb.ibers.aber.ac.uk/ accessed on 29 January 2021). Identification was based on the MS peaks to pathway algorithm [82] (tolerance = 5 ppm, reference library; *Arabidopsis thaliana*). This involved metabolites being annotated using the KEGG database, considering the following possible adducts: [M+]+, [M+H]+, [M+NH4]+, [M+Na]+, [M+K]+, [M-NH2+H]+, [M-CO2H+H]+, [M-H2O+H]+; [M−]−, [M−H]−, [M+Na−2H]−, [M+Cl]−, [M+K−2H]−. Correlations between multiple adducts of the suspected metabolite were used in the identification process.

### 4.3. Metabolomic Statistical Analysis

Statistical analysis was performed on obtained data after normalizing to % total ion count and log_10_-transformed in MetaboAnalyst 4.0 (https://www.metaboanalyst.ca accessed on 8 February 2021). Differences in the metabolomic profiles of samples were analyzed with unsupervised principal component analysis (PCA) and supervised partial least squares-discriminant analysis (PLS-DA). Significant variables were defined based on cross-validated *p*-values derived from one-way analysis of variance (ANOVA) with Bonferroni correction for false discovery rates (FDR). Multiple comparisons and post hoc analyses used Tukey’s Honestly Significant Difference (Tukey’s HSD). Fisher LSD test was used to determine which compounds varied significantly between groups at *p* < 0.05. Results were visualized in the form of hierarchical cluster analyses incorporating heat maps. All significant metabolites were mapped into metabolomic pathways based on the Kyoto Encyclopedia of Genes and Genomes (KEGG) database.

### 4.4. Exogenous Phytohormone Treatments

For phytohormone treatment studies, 100 µM ABA (Sigma, Bangalore, India), 1 mM SA (Sigma, Bangalore, India) and 500 µM methyl jasmonate (MeJA) (Sigma, Bangalore, India) were used with 0.02% Tween 20 as a wetting agent. Plants were treated with phytohormones by spraying before the inoculation with *P. capsici*. Mock-inoculated plants were treated with distilled water along with 0.02% Tween 20.

### 4.5. Quantitative Real-Time (qRT)-PCR

Gene-specific primers were designed based on *P. nigrum* transcriptome data [83] and Oligocalc was used in self-complementarity and secondary structure predictions (http://biotools.nubic.northwestern.edu accessed on 7 April 2021) (Table 1). Samples from infected and control plantlets collected for metabolomic profiling were used for further RNA extraction. Total RNA was isolated from leaf tissue of *P. capsici*-infected and mock-infected *P. nigrum* using *mir*Vana™ miRNA Isolation Kit (Thermo Fisher Scientific, Waltham, MA, USA) according to the manufacturer’s protocol. 1 μg of total RNA was reverse transcribed using a High-Capacity cDNA Reverse Transcription Kit (Applied Biosystems™, Waltham, MA, USA). RT-qPCR was performed in a 5 μL reaction mixture containing the following: 2.5 μL 2 × SYBR Master mix (Applied Biosystems™), 1 μL cDNA template (50 ng) and 0.25 uL of each forward and reverse primer (10 ρM) and made up to 5 μL with nuclease-free water. PCR amplification involved the following conditions: 40 cycles at 95 °C for 15 s and 65 °C for 15 s. Negative PCR controls (without cDNA template) were prepared to detect any contamination from cDNA. 5.8S rRNA was used as the reference gene. Three biological and three technical replicates per sample were analyzed using the Applied Biosystems 7900 HT sequence detection system (ABI). Relative quantification was analyzed by comparative CT method using 2^−ΔΔCT^ method [84].

## 5. Conclusions

Overall, this study showed that black pepper leaf metabolites were significantly affected by *P. capsici* infection. Taking our data together, non-targeted metabolomics analysis showed the metabolic changes in the control vs. *P. capsici*-infected black pepper leaf tissues. Spatial and temporal specific metabolic variations were also observed, which demonstrated that systemic and infected leaf tissues undergo differential metabolomic changes in response to infection at different time points. As a part of the host responses, black pepper plants indicated a shift in primary metabolism (amino acids, TCA, etc.) in response to infection by *P. capsici* which may ultimately benefit the pathogen. We presume that the higher levels of vit. B6 in the black pepper was one of the factors causing susceptibility to *P. capsici*. Variations in the levels of phytohormones were clearly observed in infected leaves compared to control. Defensive phytohormones are produced but the effects of these are likely to be suppressed by ABA production. This was in turn confirmed by the qPCR analysis where reduced expression of *ICS1* and *AOS*, the precursors of SA and JA respectively, was observed. However, exogenous elicitors can maintain or boost the SA/JA effects and could represent a method of reducing quick wilt disease of pepper in the field.

## Figures and Tables

**Table 1:** Primer sequences used in RT-qPCR analyses.

Gene	Forward Primer	Reverse Primer
*PPOX*	CACTCGTCTTTCTTTCAG	TGCGACTTCTACTATCTG
*ICS1*	GAGTCTGAATTTGCGGTAGG	CCAACTCCTCCCACTCTAA
*AOS*	CGGCTCTACGACTTCTTCTA	AGAAGGCGAAGACAAGGT

## Data Availability

The data that support the findings of this study are openly available in https://www.ncbi.nlm.nih.gov/sra/?term=SRA050094 and at National Center for Biotechnology Information.

## Supplementary Materials

The following are available online at https://www.mdpi.com/article/10.3390/ijms222111433/s1.
