# Supplementary material for: Non-Targeted Metabolite Profiling Reveals Host Metabolomic Reprogramming during the Interaction of Black Pepper with Phytophthora capsici
Source: Int J Mol Sci. 2021 Oct 22;22(21):11433. doi: 10.3390/ijms222111433 (PMC8583951; doi:10.3390/ijms222111433)
Supplement: Supplementary file 1 [file ijms-22-11433-s001.zip › ijms-1376018-supplementary.pdf]

Non-targeted metabolite profiling reveals host metabolomic reprogramming during the interaction of black pepper with *Phytophthora capsici*

[1]Divya Kattupalli., [2]Artur Pinski., [1]Sweda Sreekumar., [1]Aswathi U., [3]Aiswarya Girija., [3]Manfred Beckmann., [3]Luis A. J. Mur*✉ and [1]Soniya E.V.*✉

Supplementary Figures

Figure S1

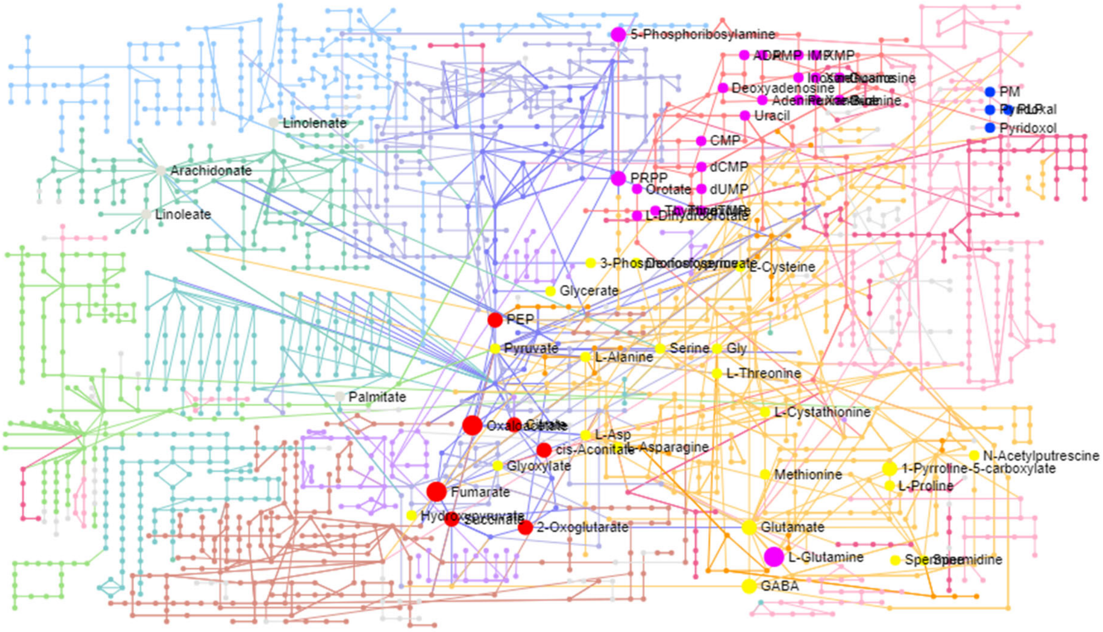

Figure S1: The major sources of variation in the metabolome Black Pepper (*Piper nigrum* L.) leaves challenged with *Phytophthora capsici*mapped on to the KEGG global metabolite pathway.

Metabolites were colour coded based on being classified as being involved in amino acid metabolism

([alanine, aspartate, glutamate], [glycine, serine, threonine], [cysteine, methionine], [arginine and proline]), bioenergy(citrate and pyruvate), nucleotide metabolism(purines and pyrimidines), vitamin B6 metabolism and the biosynthesis of unsaturated fatty acids.

Figure S2

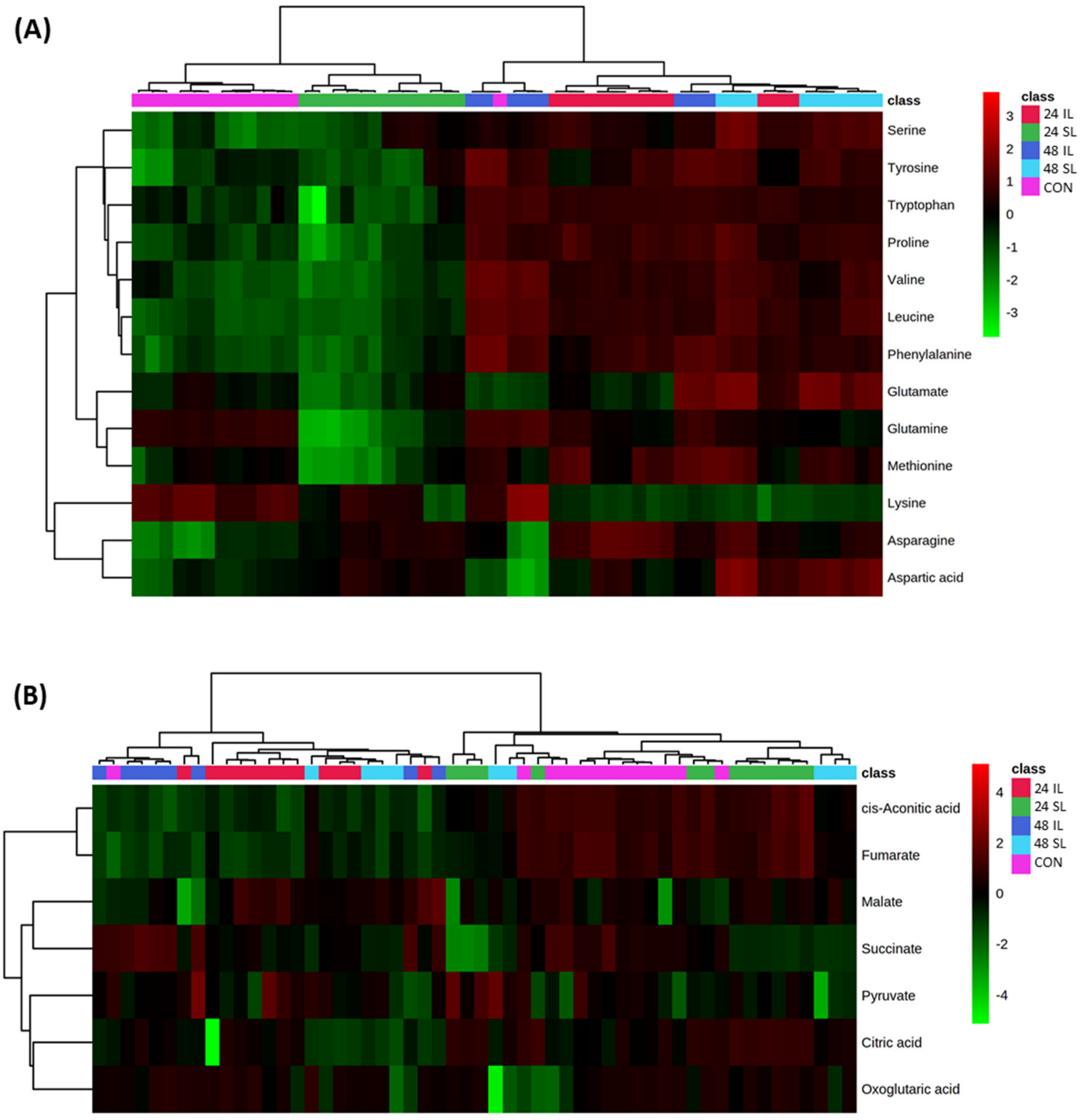

Figure S2: Heat map showing the changes in **(A)** amino acid and **(B)** citric acid cycle metabolism in Black Pepper (*Piper nigrum* L.) leaves challenged with *Phytophthora capsici*.

Amino acid variation in leaves at the site of infection (i-L) and in the leaf immediately above the infection site (i-SL, systemic leaf) at 24 and 48 h after challenge and controls (CON). The relative accumulation of each is displayed using a heat map.
